# Decent work, work engagement, and turnover intention among registered nurses: a cross-sectional study

**DOI:** 10.1186/s12912-023-01662-6

**Published:** 2024-01-10

**Authors:** BowenXue, Yaping Feng, Yihui Zhao, Xin Li, Yang Yang, Jingxuan Zhang, Yu Zhang, Zhiguo Hu, Hong Luo

**Affiliations:** 1 Affiliated Mental Health Center & Hangzhou Seventh People’s Hospital, Zhejiang University School of Medicine, Hangzhou, 310007 Zhejiang China; 2 Hangzhou Normal University, Hangzhou, 310007 Zhejiang China; 3 Affiliated Hospital of Hangzhou Normal University, Hangzhou, 310015 Zhejiang China

**Keywords:** Decent work, Work engagement, Turnover intention, Nurses, Mediation analysis

## Abstract

**Background:**

Nurses face substantial career challenges arising from global pandemics, economic crises, and their roles in conflict-ridden areas. In this context, the rights of nurses pertaining to decent work, such as freedom, fairness, safety, and dignity, are not adequately safeguarded. This study examines decent work status among Chinese nurses and its links to demographics, work engagement, and turnover intention.

**Methods:**

A cross-sectional study design was used following STROBE guidelines. Through a convenient sampling method, a total of 476 nurses were surveyed. These participants were drawn from three esteemed tertiary Grade A hospitals in Hangzhou, with data collection spanning from June to August in 2023. We used a comprehensive set of assessment instruments, encompassing an evaluation of demographic characteristics, the Decent Work Perceptions Scale (DWPS), the Utrecht Work Engagement Scale (UEWS), and turnover intention questionnaire. Bootstrapping procedures were used to ensure the robustness and reliability of the model.

**Results:**

The study revealed that nurses' perceptions of decent work significantly impacted work engagement (*β* = 0.603, *p* < 0.001) and turnover intention (*β* = -0.275, *p* < 0.001). Work engagement operated as a mediator between decent work and turnover intention, decreasing the likelihood of nurses leaving their positions (*β* = -0.062, *p* < 0.001). Factors such as age, years of working experience, professional title, job category, and attendance at professional conferences significantly influenced nurses' perceptions of decent work (all *p* < 0.05).

**Conclusions:**

This study examines factors affecting decent work among nurses and explores its connection with work engagement and the intention to leave. Despite limitations (sample, social desirability bias), the study offers valuable insights for nursing practice. This suggests managers improve decent work for young nurses through rational shift schedules and continuous education. Policymakers should consider adjusting nursing policies for better employment conditions.

**Supplementary Information:**

The online version contains supplementary material available at 10.1186/s12912-023-01662-6.

## Introduction

In recent years, the global COVID-19 pandemic and localized economic and wartime crises have placed many nurses in a precarious position. They confront inadequacies in welfare protection, a decline in social status, and challenging working conditions. Notably, there is a discernible shift in the professional landscape for nurses both preceding and following the pandemic. The average score reflecting nurses' likelihood to leave increased from 13.24 to 15.54 [1], suggesting a heightened inclination toward professional disengagement. What is even more concerning is the inadequate protection of nurses' working conditions [2], including extended working hours [3], high job-related stress levels [4], and workplace violence [5]. It is evident that traditional research perspectives often confine themselves to specific manifestations of professional challenges faced by nurses, overlooking considerations related to dignity, rights, fairness, and overall development in the workplace. The United Nations Sustainable Development Goal (SDG) 8 is explicitly focused on labor and is titled "Decent Work and Economic Growth." It highlights the importance of promoting sustained, inclusive, and sustainable economic growth and providing full and productive employment along with decent work for all [6]. However, existing research indicates a limited focus on the concept of decent work among nurses, particularly in exploring the relationships of associated variables and understanding influencing factors. Therefore, prioritizing the concept of 'decent work' and its associated variables among nurses, alongside an examination of the influencing factors, is an essential imperative for advancing the sustainable development of health.

## Background

Within the context of the rapidly evolving landscape of the nursing profession, the concept of 'decent work' emerges as a crucial notion. encompassing equitable income, social security, safe working conditions, opportunities for personal development and social integration, as well as the freedom to express opinions and raise concerns in the workplace [7, 8]. In accordance with the International Labor Organization (ILO), it is evident that decent work embodies a fusion of four fundamental values: freedom, fairness, safety, and human dignity, encompassing a range of broader principles related to equitable and dignified employment [9]. Numerous surveys and studies focusing on nurses and other professional groups across various regions consistently indicate that decent work correlates with individual professional resilience, teamwork, job satisfaction, work engagement, and occupational burnout [10–12]. These findings underscore the comprehensive impact of decent work on both individuals and organizations. However, previous research has indicated that nurses often perceive the concept of decent work slightly below the average level, and their perception is influenced by characteristics specific to the nursing work environment [13]. Within this complex interplay, it becomes evident that the concept of decent work affects work engagement among nurses [14].

Work engagement refers to a state characterized by a positive and fulfilling state of mind related to work, marked by vitality, dedication, and absorption [15]. Vitality represents a sense of professional psychological resilience and energy levels during work. Dedication denotes a strong commitment to work, where individuals experience the meaning, challenge, inspiration, pride, and enthusiasm in what they do. Absorption refers to the feeling of being focused and completely engrossed in their work. When individuals perceive that they have decent work conditions, including fair compensation, job security, and opportunities for personal growth, they are more likely to feel satisfied and secure in their work environment [16]. Previous research has demonstrated that nurses' work engagement is a key driver of nursing quality and can predict nurses' job satisfaction [17], nurse‒patient relationships [18], and turnover intention [19, 20]. When an individual's level of work engagement is low, they are more likely to disengage from their work roles, consequently affecting job performance and even leading to turnover intention.

Turnover intention refers to an individual's inclination to leave their current job and seek other employment opportunities [21], often serving as a strong predictor of actual turnover behavior [22]. While nurses with high turnover intentions may not necessarily act upon these intentions, research has shown associations between high turnover intentions, absenteeism, and missed nursing care [20]. Additionally, studies have found that "decent work," which includes factors such as equitable compensation, favorable working conditions, opportunities for leisure, access to medical benefits, and alignment with personal values, constitutes a more comprehensive construct that effectively predicts employees' turnover intentions [16]. Therefore, gaining insights into this process is essential for addressing high turnover intentions among nurses and, consequently, patient safety concerns.

The psychology of working theory (PWT) provides a framework for understanding the relationships between decent work, work engagement, and turnover intention [23]. According to the PWT, decent work is subject to the influence of environmental variables, such as economic constraints and marginalization. Moreover, there are psychological variables, such as decision-making autonomy and career adaptability, which act as mediating variables. Concurrently, personality traits, such as proactivity, function as moderating mechanisms. This comprehensive framework has been substantiated by multiple research studies [24, 25]. On the other hand, previous research has identified key elements of decent work, including a safe working environment, income, leisure time, and values. These factors have been found to positively predict individuals' job satisfaction while negatively predicting turnover intentions [26].

While there has been some attention directed toward the concept of decent work among nurses, it is noteworthy that the field of nursing research currently lacks comprehensive investigations into the concept of decent work among registered nurses, as well as the influential factors that contribute to it. Moreover, there has been limited attention to the relationships between decent work, work engagement, and turnover intention. Therefore, this study focuses on clinically registered nurses as the study population. The primary objectives of this research are to assess the current status of decent work within this specific group, conduct a comprehensive analysis of the multifaceted factors influencing decent work, and investigate the relationships between decent work, work engagement, and turnover intention. Ultimately, our overarching goal is to promote the positive and sustainable development of clinical nursing.

## Methods

### Design

This research was conducted as a cross-sectional study, adhering to the guidelines provided by the Strengthening the Reporting of Observational Studies in Epidemiology (STROBE) checklist (see Appendix S1).

### Participants

In this study, considering the limitations of time and resources, a convenience sampling method was employed to survey nurses from three tertiary Grade A hospitals in Hangzhou, Zhejiang Province, China. The selection of these hospitals was based on their similar scale and working environments. The objective of this decision was to ensure research validity while maximizing internal validity. Data collection took place between June 2023 and August 2023. The sample size calculation was performed using the G*Power program (version 3.1). Using the linear multiple regression approach, the study incorporated a moderate effect size of 0.15 [27]. This decision aimed to balance statistical power and the necessary sample size, avoiding overly optimistic expectations while ensuring the ability to detect meaningful results. In setting the parameters for the study, we defined the α level as 0.05 to control the acceptable Type I error rate. Simultaneously, we aimed for a desired power (1-β error probability) of 0.95, highlighting our emphasis on reducing Type II errors. The study involved 23 variables, comprising 12 sociodemographic characteristics and 11 scale-associated dimensions. The calculated minimum sample size was 234 nurses. Factoring a potential 10% rate of invalid questionnaires, a minimum of 257 nurses were deemed necessary for participation in this study.

### Inclusion and non-entry criteria determination

The inclusion and non-entry criteria for this study were meticulously developed through collaborative discussions within the research team. The comprehensive details are as follows:

The inclusion criteria for this study were as follows: (a) nurses with a minimum of one year of clinical experience, (b) registered nurses, and (c) voluntary participation in the study.

The non-entry criteria were as follows: (a) nursing interns or trainees, (b) nurses who were not on duty during the survey period, (c) nonclinical nurses, such as those working in medication distribution centers, and (d) nurses who declined to participate in the study.

Ultimately, the research encompassed 500 nurses out of a target population of 1640 nurses. A set of 500 questionnaires was distributed, and all 500 were successfully collected. Following the exclusion of 27 questionnaires due to abnormal responses (e.g., 12121212, 11111111), 476 questionnaires were deemed valid, resulting in an effective response rate of 95.2%.

### Data collection

The data collection process for this study employed a combination of online and offline methods. First, project team members contacted the heads of nursing departments in various hospitals via phone, email, and other means to extend invitations to participate in the research. Upon receiving approval from the nursing department heads, the research team distributed both paper-based questionnaires and electronic questionnaire links to the nurses.

Subsequently, the principal investigator conducted uniform training for all data collectors to ensure a clear understanding of the research objectives and content. Particular emphasis was placed on respecting the rights of participating nurses and underscoring the significance of obtaining informed consent. During the data collection phase, researchers provided detailed explanations to participating nurses regarding the study's purpose, expected outcomes, and the importance of their involvement. It was explicitly highlighted that participation in this study was entirely voluntary, and participants had the option to withdraw at any point without facing any negative consequences. Additionally, the research's confidentiality and data protection measures were clearly articulated, ensuring full respect for participants' privacy.

All participating nurses willingly agreed to participate in the study, with the freedom to choose their preferred method of responding to the questionnaires. The questionnaires incorporated standardized instructions to introduce the data collection requirements and guidelines to the participants. Upon completion of the questionnaires, they were immediately reviewed on-site to ensure data validity and completeness. Finally, data entry was carried out by two individuals, cross-verified for accuracy to ensure precise data recording.

### Instruments

#### Demographic characteristics

The demographic characteristics included gender, age, education background, marital status, number of children, years of working, professional title, department, leadership roles, type of employment, monthly night shift frequency, and annual participation in external educational activities.

#### Decent work perceptions

The Decent Work Perceptions Scale (DWPS), initially developed by Chinese scholars, has undergone meticulous assessments of its reliability and validity [28]. It consists of five distinct dimensions, encompassing a total of 16 items. These dimensions include Work Rewards (comprising 4 items), Work Position (containing 3 items), Work Atmosphere (including 3 items), Work Development (consisting of 3 items), and Work Recognition (comprising 3 items). Respondents utilize a 5-point Likert scoring system, with response options ranging from "Strongly Disagree" to "Strongly Agree," correspondingly scored from 1 to 5. The cumulative score on this scale falls within the range of 16 to 80 points, with higher scores denoting a more robust perception of decent work. It is noteworthy that the DWPS exhibited a Cronbach's α coefficient of 0.939 in this study.

#### Work engagement

The Work Engagement Scale used in this study was adapted from the Utrecht Work Engagement Scale (UEWS) developed by Schaufeli et al. [29]. The Chinese version of the scale, which was translated and revised by Zhang Yi-wen [30], was used to assess the work engagement status of nurses. The UEWS comprises three dimensions: vigor, dedication, and absorption, totaling 15 items. Specifically, the 'vigor' dimension consists of six items, the 'dedication' dimension includes four items, and the 'absorption' dimension comprises five items. Each item is rated on a 7-point Likert scale, ranging from 'never' to 'always', with scores ranging from 0 to 6. The total score on the scale falls within the range of 0 to 90. In this study, the Cronbach's α coefficient for the scale was calculated to be 0.930.

#### Turnover intention

The Chinese version of the turnover intention scale(TIS) is based on the instrument originally developed and validated by Michaels and Spector [31]. It has been subsequently translated, adapted, and validated by Chinese researchers for the purpose of assessing nurses' turnover intentions [32, 33]. The questionnaire comprises three dimensions, each consisting of two items: Turnover Intention I, representing the inclination to resign from the current job; Turnover Intention II, indicating the desire to seek alternative job opportunities; and Turnover Intention III, reflecting the readiness for a new job. Participants employ a 4-point scale to rate each item, with 1 indicating "Never" and 4 indicating "Always." The total score ranges from 6 to 24 points. The severity of turnover intention is categorized into four levels: 0-6, 7-12, 13-18, and 19-24, corresponding to low, moderate, moderate to high and high degrees of intention to leave, respectively, with higher scores indicating a stronger intention to leave [34, 35]. The reliability of this scale has been consistently demonstrated in multiple studies, with Cronbach's α coefficients ranging from 0.75 to 0.81 [36]. The Cronbach's α coefficient for this study's questionnaire is 0.907.

#### Data analysis

The statistical analysis was conducted using IBM SPSS Statistics, version 26.0. Categorical variables were described in terms of frequencies and percentages. For univariate analysis, the appropriate statistical test was selected based on the data type. Specifically, we employed the t test for comparing two groups of data and utilized one-way analysis of variance (ANOVA) for comparing multiple groups of data. Within the variables, because "department" and "marital status," among others, were considered unordered categorical variables, we included them in the model after creating dummy variables. To explore the relationships between decent work, work engagement, and turnover intention, Spearman's correlation analysis was used. Variables with p values less than.05 in both univariate and correlation analyses were subsequently included in the multiple linear regression analysis. A significance level of p <.05 was deemed statistically significant. We conducted mediation analysis using Hayes' PROCESS Macro [37].

## Results

Table 1 provides an overview of the demographic and employment characteristics of the nursing staff in the study. The majority of participants were female (95.6%), with a small representation of males (4.4%). Age categories showed a distribution across the four groups, with the largest proportion falling in the ≥36 age range (38.7%). Educational backgrounds predominantly included individuals with bachelor's degrees (95.8%), while marital status indicated that a substantial portion of participants were married (71.0%). Concerning the number of children, approximately one-third had none (33.2%), and two-thirds had 1-3 children (66.8%). Work experience varied, with a significant number having 5-15 years of experience (57.8%). Senior nurses were the most common professional title (52.1%), and the majority worked in Medicine or Surgery departments (62.6%). Leadership roles were held by 9.0% of participants, while the majority did not (91.0%). Permanently employed nurses constituted 66.2%, and monthly night shift frequencies were diverse. The majority attended fewer than two annual conferences (81.7%).

**Table 1:** Sociodemographic characteristics of the sample (*N* = 476)

**Demographics**	***n(%)***	**Decent Work**			**Work Engagement**			**Burnout**		
		**M ± SD**	***F/t***	***p***	**M ± SD**	***F/t***	***p***	**M ± SD**	***F/t***	***p***
**Gender**			-1.889	0.059		-1.897	0.058		1.556	0.120
Male	21(4.4)	49.10±10.76			44.81±17.25			15.33±3.45		
Female	455(95.6)	53.56±10.57			51.64±16.08			14.01±3.83		
**Age**			6.187	<0.001		5.777	<0.001		33.693	<0.001
≤25	40(8.4)	47.73±14.66			44.83±21.54			16.30±3.80		
26-30	99(20.8)	53.07±8.47			49.30±14.91			15.71±3.42		
31-35	153(32.1)	52.01±9.19			49.87±15.24			14.75±3.10		
≥36	184(38.7)	55.86±11.08			55.07±15.50			12.14±3.73		
**Educational Background**			1.086	0.338		0.734	0.480		2.563	0.078
Short-cycle Courses	16(3.4)	52.56±8.34			48.63±9.67			12.06±4.22		
Bachelor's degree	456(95.8)	53.32±10.70			51.36±16.33			14.15±3.80		
Master's degree or above	4(0.8)	61.00±3.37			59.50±18.88			12.75±2.22		
**Marital status**			0.618	0.539		1.850	0.158		13.088	<0.001
Married	338(71.0)	53.02±10.82			52.13±15.77			13.58±3.85		
Unmarried	122(25.6)	54.11±10.03			48.93±17.34			15.55±3.37		
Others	16(3.4)	54.75±10.64			52.94±14.25			13.13±3.70		
**Number of children**			-0.089	0.928		-2.717	0.007		5.725	<0.001
0	158(33.2)	53.30±10.29			48.50±16.08			15.39±3.41		
1-3	318(66.8)	53.39±10.78			52.75±16.06			13.41±3.85		
**Years of Working**			5.180	0.002		4.032	0.008		35.374	<0.001
< 5	42(8.8)	54.74±10.31			48.60±17.51			15.29±3.04		
5~15	275(57.8)	51.76±9.84			49.69±15.80			15.04±3.48		
16~25	100(21.0)	55.55±12.51			54.62±16.67			13.12±3.84		
>25	59(12.4)	56.10±9.64			55.39±14.80			10.25±2.93		
**Professional title**			5.766	<0.001		4.753	0.003		34.842	<0.001
Registered nurses	15(3.2)	55.93±9.60			45.87±16.91			14.40±5.19		
Senior nurse	248(52.1)	51.85±10.09			49.63±16.12			15.41±3.27		
Supervisor nurses	178(37.4)	54.12±11.34			52.62±16.27			12.98±3.68		
Cochief nurses or above	35(7.4)	59.03±8.40			59.23±12.84			9.94±2.75		
**Department**			-1.126	0.261		0.475	0.635		1.839	0.067
Medicine or surgery	289(62.6)	52.94±10.75			51.61±15.84			14.32±3.77		
Special departments	178(37.4)	54.07±10.35			50.88±16.76			13.65±3.88		
**Leadership roles**			6.413	<0.001		4.261	<0.001		-7.715	<0.001
Yes	43(9.0)	62.86±9.42			61.19±14.53			10.02±3.04		
No	433(91.0)	52.42±10.26			50.36±16.01			14.47±3.66		
**Job category**			3.729	<0.001		2.381	0.018		-6.338	<0.001
Permanently employed	315(66.2)	54.64±10.68			52.59±16.03			13.30±3.74		
Temporarily employed	161(33.8)	50.86±10.03			48.88±16.23			15.56±3.54		
**Monthly night shift frequency**			14.830	<0.001		13.845	<0.001		55.522	<0.001
<5	179(37.6)	56.33±10.73			56.21±15.76			11.96±3.48		
5~7	135(28.4)	53.16±9.92			48.94±14.98			14.95±3.61		
>7	162(34.0)	50.25±10.16			47.96±16.36			15.67±3.25		
**Annual conference**			-6.475	<0.001		-4.383	<0.001		6.928	<0.001
2<	389(81.7)	51.93±10.16			49.83±15.93			14.61±3.62		
≥2	87(18.3)	59.75±10.27			58.08±15.60			11.62±3.76		

Special departments: Including emergency, operating room, *ICU* Interventional center, hemodialysis, and other departments

### The scores of decent work, work engagement and turnover intention

The study participants reported an average score of 53.36 ± 10.61 for decent work, 51.34 ± 16.17 for work engagement, and 14.07±3.82 for turnover intention. The specific scores for each dimension can be found in Table 2.

**Table 2:** Descriptive statistics for the decent work, work engagement, and turnover intention

**Variables**	**Minimum**	**Maximum**	**Scores (Mean ± SD)**
**Work Rewards**	4	20	12.24 ± 3.20
**Work Position**	3	15	9.71 ± 2.32
**Work Atmosphere**	3	15	11.49 ± 2.39
**Work Development**	3	15	10.51 ± 2.42
**Work Recognition**	3	15	9.41 ± 2.40
**Decent work**	16	80	53.36 ± 10.61
**Vigor**	0	36	21.47 ±6.65
**Dedication**	0	24	13.01 ±4.59
**Absorption**	0	30	16.85 ±5.65
**Work engagement**	0	90	51.34 ±16.17
**Turnover intention I**	2	8	4.42 ± 1.49
**Turnover intention II**	2	8	4.49±1.44
**Turnover intention III**	2	8	5.16±1.36
**Turnover intention**	6	24	14.07±3.82

### Influencing factors of decent work

The results of a multiple regression analysis concerning the concept of decent work are presented in Table 3. The variables accounted for 14.6% of the variance in decent work. Notably, age (≤25 vs. ≥36), years of working (<5 vs. 5~15), monthly night shift frequency (<5 vs. >7), and annual conference (2< vs. ≥ 2) emerged as significant influencing factors affecting decent work.

**Table 3:** Correlation of decent work, work engagement and turnover intention (*N* = 476)

**Variables**	**1**	**2**	**3**
**Decent work**	**1**		
**Work engagement**	0.603**	**1**	
**Turnover intention**	-0.446**	-0.449**	**1**

^**^p<0.001; 1= Decent work; 2= Work engagement; 3= Turnover intention

### Correlations among decent work, work engagement and turnover intention

The findings of Pearson's correlation analysis examining the associations among decent work, work engagement, and turnover intention are presented in Table 4. It was observed that work engagement demonstrates a positive correlation with decent work, while turnover intention exhibits a negative correlation with decent work.

**Table 4:** Regression analysis results

**Variables**	***β***	***SE***	***t***	***p***
**Age (≤25 vs. ≥36)**	**-12.030**	**2.724**	**-4.416**	**<0.001**
**Age (26~30 vs. ≥36)**	**-1.733**	**2.049**	**-0.845**	**0.398**
**Age (31~35 vs. ≥36)**	**-1.846**	**1.747**	**-1.057**	**0.291**
**Years of Working (<5 vs.5~15)**	**7.995**	**2.192**	**3.647**	**<0.001**
**Years of Working (16~25 vs.5~15)**	**0.068**	**1.715**	**0.040**	**0.968**
**Years of Working (≥25 vs.5~15)**	**-0.713**	**2.215**	**-0.322**	**0.748**
**Professional title** (registered nurses **vs.** senior nurse)	**4.908**	**2.834**	**1.732**	**0.084**
**Professional title** (supervisor nurses **vs.** senior nurse)	**-2.328**	**1.459**	**-1.596**	**0.111**
**Professional title** (cochief nurses or above **vs.** senior nurse)	**-1.064**	**2.622**	**-0.406**	**0.685**
**Job category** (permanently employed **vs.** temporarily employed)	**-2.100**	**1.158**	**-1.813**	**0.070**
**Monthly night shift frequency (<5 vs.5~7)**	**-1.128**	**1.420**	**-0.794**	**0.427**
**Monthly night shift frequency (<5 vs. >7)**	**-3.140**	**1.412**	**-2.223**	**0.027**
**Annual conference (2<vs. ≥ 2)**	**6.126**	**1.353**	**4.527**	**<0.001**
**R**^**2**^	**0.169**			
**Adjusted R**^**2**^	**0.146**			

### The mediating role of work engagement in the relationship between decent work and turnover intention

The PROCESS macro analysis revealed several significant associations among the variables in our study. Specifically, we found that decent work significantly predicts turnover intention (β = -0.275, p < 0.001), explaining approximately 19.9% of the total variance in turnover intention. Furthermore, decent work significantly predicts work engagement (β = 0.603, p < 0.001), accounting for approximately 36.3% of the variance in work engagement. Work engagement, in turn, significantly predicts turnover intention (β = -0.283, p < 0.001), serving as a mediator in the relationship between decent work and turnover intention (β = -0.062, p < 0.001) and explaining approximately 25.0% of the variance in turnover intention. These results are supported by a 95% bootstrap confidence interval of -0.086 to 0.036 (refer to Table 5).

**Table 5:** The mediating role of work engagement in decent work and turnover intention

**Effect**	**Variable**	**Adjusted R**^**2**^	**Coefficient**	**SE**	***p***	**LLCI**	**ULCI**
Direct effect	Decent Work → UEWS	0.363	0.603	0.056	<0.001	0.810	1.029
	Decent Work → TIQ	0.199	-0.275	0.018	<0.001	-0.134	-0.064
	UEWS→TIQ		-0.283	0.012	<0.001	-0.090	-0.044
Indirect Effect (UEWS)	Decent Work →UEWS→TIQ	0.250	-0.062	0.013	<0.001	-0.086	-0.036
Total Effect			-0.160	0.015	<0.001	-0.190	-0.131

## Discussion

In this study, through a survey of clinical registered nurses, it was found that the average score for decent work among registered nurses was 53.36 ± 10.61. This finding is similar to previous investigations into the concept of decent work among Chinese registered nurses [38]. Sociodemographic factors (age, years of working, professional title, job category, and annual conference) were identified as the primary factors influencing nurses' perception of decent work. Work engagement serves as a mediator between decent work and turnover intention among nurses.

In this study, the results indicate that nurses aged 25 or younger, those with less than 5 years of working experience, and registered nurses tend to score lower on measures of decent work. On one hand, this observation may be linked to the distinct work values and employment preferences of the millennial generation, highlighting a shift in priorities beyond merely seeking high income [39]. Millennials often prioritize factors such as having sufficient free time and aligning with their personal life values in their work [40]. On the other hand, these trends may be attributed to the relatively lower remuneration [41], lack self-confidence, sufficient work experience and problem-solving skills [42], and the young nurses' lack of firmly established professional identity [43], which are commonly associated with younger nurses and may compromise their perception of work decency. Consequently, healthcare institutions should not only assist young nurses in finding fulfillment in their work but also provide them with adequate time to enjoy their personal lives [40].

The results of regression analyses revealed that the perception of decent work among nurses is influenced not only by demographic factors such as age and professional title but also by job category and attendance at annual conferences. Temporary nurses, in particular, may perceive their work as lacking in decency due to the potential instability associated with their career [44]. This observation aligns with the tenets of the PWT, emphasizing the importance of survival needs [23]. In light of these findings, healthcare organizations are urged to take proactive measures to alleviate concerns about the future career prospects of temporary nurses. For instance, implementing comprehensive career development plans or offering pathways to transition into more stable positions could be beneficial. Furthermore, nurses who actively participate in professional academic conferences may experience enhanced professional recognition, knowledge enrichment, expanded interpersonal networks, and heightened professional self-esteem and self-confidence [45]. Recognizing the positive impact of conference participation, healthcare organizations can leverage this enthusiasm for continuous learning. Facilitating ongoing opportunities for continuing education and skills development not only aligns with the nurses' pursuit of knowledge but also fosters a heightened sense of job decency.

Additionally, our study reveals that there is a strong association between work engagement and turnover intention. This suggests that when nurses are fully engaged in their work, they are less likely to contemplate leaving their current positions. This is consistent with the findings of several previous studies [16, 40]. High levels of work engagement manifest in nurses displaying a strong sense of responsibility and professional commitment [46], extending beyond a mere employment relationship. They view their work as a mission, leading them to address professional challenges with a proactive attitude and diligent work rather than resorting to avoidance or seeking new positions [46]. Moreover, the passion and commitment exhibited by nurses in their professional roles enhance their personal job satisfaction and alleviate their inclination to seek alternative employment [47]. Given these outcomes, healthcare institutions should cultivate a positive work environment that fosters nurses' work engagement and diminishes turnover intentions. Additionally, it is crucial to establish suitable incentive mechanisms that guarantee acknowledgment of nurses' efforts. Implementation of flexible work arrangements is equally important to address their work-life needs [48]. These measures contribute not only to the improvement of healthcare service quality and efficiency but also to the formation of a resilient and effective nursing team within healthcare organizations.

The findings of this study reveal a positive correlation between decent work and work engagement, and the perception of decent work among nurses significantly predicts their level of work engagement. Nurses who view their work as decent are more inclined to invest time and effort in their professional roles. This finding aligns with prior research within the academic personnel context [9]. On the one hand, when individuals perceive their work as decent, they are more likely to experience a sense of safety, satisfaction, and achievement in their work [16]. On the other hand, there is also evidence suggesting that elements of decent work, such as favorable working conditions and job security, are related to work engagement [49]. When working conditions are conducive and fulfill fundamental needs for survival, social connections, and societal determinants, individuals are more inclined to invest in their tasks and responsibilities [50]. This underscores the importance for managers to prioritize and implement measures to ensure nurses have a decent working environment. For example, providing fair and equitable compensation, a safe working environment, good career development opportunities, and ample social security [51]. Additionally, efforts should be made to enhance the image and recognition of the nursing profession, boosting nurses' professional identity and self-esteem consequently improving their perception of decent work.

Finally, our research emphasizes the correlation between decent work, work engagement, and turnover intention, highlighting the mediating role of work engagement in the association between decent work and turnover intention. This implies that decent work contributes, at least partially, to reducing turnover intention by impacting work engagement. This aligns with prior research results [14]. In prior research, it was established that equitable remuneration, secure working conditions, chances for personal growth, and the presence of decent labor conditions, including freedom of expression, have a direct impact on individual job satisfaction and their perception of work [16]. When individuals perceive their work as decent, it signifies that their work environment meets their fundamental needs, including fair treatment, respect, and safety assurances [52]. In a contented work environment, individuals are motivated, gain recognition for their work, and develop emotional commitment to the organization [53]. This positive relationship further emphasizes the crucial role of work engagement in diminishing turnover intention [54]. This suggests that healthcare institutions can proactively nurture and sustain employees' work engagement by providing ongoing training, establishing effective communication channels, and implementing reward systems, thus effectively reducing turnover intentions and enhancing work efficiency and service quality.

### Implication

In conclusion, nursing management should take into account various factors such as age, experience, job category, and career development when establishing a work environment that promotes decency. Particularly for younger nurses, it is essential to implement structured guidance programs, providing them with ample support and a systematic career development pathway. Additionally, tailored career development plans, including personalized training programs and mentorship systems, should be designed for nurses at different career stages to ensure promising career prospects [55]. Moreover, ensuring competitive salary packages and job security is crucial for reducing nursing turnover and safeguarding the key elements of decent work, especially for temporary nursing staff [56]. Such strategies not only address their concerns about future career prospects but also effectively enhance their perception of work decency. Therefore, management institutions should prioritize the formulation of specific benefits and incentive mechanisms for temporary nurses. Simultaneously, nursing managers should recognize the value of continuous education and professional networking, actively encouraging nurses of all age groups to participate in professional conferences for skill enhancement, staying informed about industry trends, and strengthening professional identity [57]. Finally, by focusing on measures such as supporting nurses' work engagement, providing resources, creating a positive work environment, offering motivation and recognition, promoting work-life balance, and fostering a sense of participation, nursing turnover rates can be reduced, leading to improved nursing quality and patient satisfaction [58].

### Limitations

**Thi**s study has several limitations that should be acknowledged. First, the survey was conducted in three public hospitals in the Hangzhou region, which may limit the generalizability of the findings to nurses in different healthcare settings, such as those in private hospitals or other regions. Therefore, future research could benefit from conducting surveys in multiple centers with larger samples to enhance the representativeness and generalizability of the results. Second, the adoption of a cross-sectional design in this study offers a snapshot of the relationships investigated. However, it impedes the establishment of causal relationships between variables, and future research can use longitudinal studies to explore dynamic changes in nurses' careers, investigating the lasting effects of decent working conditions on work engagement and turnover intention. Last, the study recognizes the potential influence of social desirability bias on nurses' responses. Given the nature of the topic, there is a possibility that nurses may have provided responses they deemed socially acceptable, leading to an overestimation of decent work perceptions. To address this in future research, careful consideration of survey design and the implementation of techniques to minimize social desirability bias, such as indirect questioning, could be explored.

## Conclusion

In conclusion, our study delves into decent work among Chinese registered nurses, revealing key influences on their work engagement and turnover intention. Emphasizing the significance of decent work, especially for younger nurses, and highlighting work engagement as a crucial mediator, our findings contribute to nursing management. For administrators, our research offers a roadmap for targeted measures to enhance nurses' perception of decent work, fostering a resilient and engaged nursing workforce.

### Supplementary Information

**Additional file 1.** 

## Data Availability

The data that support the findings of this study are available from the corresponding author on reasonable request.

## Abbreviations

STROBE: Strengthening the Reporting of Observational studies in Epidemiology (STROBE)
ILO: International Labor Organization
SPSS: Statistical Product and Service Solutions
SD: Standard Deviation
COVID-19: Corona Virus Disease-19
PWT: Psychology of Working Theory
